# Newborn Care Practices among Adolescent Mothers in Hoima District, Western Uganda

**DOI:** 10.1371/journal.pone.0166405

**Published:** 2016-11-17

**Authors:** Lydia Kabwijamu, Peter Waiswa, Vincent Kawooya, Christine K. Nalwadda, Monica Okuga, Elizabeth L. Nabiwemba

**Affiliations:** 1 Makerere University, College of Health Sciences, School of Public Health, Kampala, Uganda; 2 Centre of Excellence for Maternal and Newborn Health, Makerere University College of Health Sciences School of Public Health, Kampala, Uganda; 3 Global Health, Department of Public Health Sciences, Karolinska Institutet, Stockholm, Sweden; Institute of Tropical Medicine, JAPAN

## Abstract

**Introduction:**

Adolescent childbearing remains a major challenge to improving neonatal mortality especially in Sub Saharan countries which are still struggling with high neonatal mortality rates. We explored essential newborn care practices and associated factors among adolescent mothers in Western Uganda.

**Methods:**

Data were collected among 410 adolescent mothers with children aged one to six months in Hoima district. Three composite variables (appropriate neonatal breastfeeding, cord care and thermal protection) were derived by combining related practices from a list of recommended newborn care practices. Logistic regression analysis was conducted to identify factors independently associated with practice of essential newborn care.

**Results:**

Appropriate newborn feeding, optimal thermal protection and dry cord care were practiced by 60.5%, 67.2% and 31% of adolescent mothers respectively. Independent predictors’ of cord care were: knowledge of cord care (AOR 5.34, 95% CI (1.51–18.84) and having delivered twins (AOR 0.04, 95% CI (0.01–0.22). The only predictor of thermal care was knowledge (AOR 25.15, 95% CI (7.01–90.20). Staying in a hospital for more than one day postpartum (AOR 2.45, 95%CI (1.23–4.86), knowledge of the correct time of breastfeeding initiation (AOR 14.71, 95% CI (5.20–41.58), predicted appropriate neonatal feeding, whereas; adolescent mothers who had had a caesarean delivery (AOR 0.19, 95% CI (I 0.04–0.96) and a male caretaker in the postnatal period (AOR 0.18, 95% CI (0.07–0.49) were less likely to practice the recommended newborn feeding.

**Conclusion:**

Sub optimal essential newborn care practice was noted especially suboptimal cord care. Adolescent mothers should be a focus of strategies to improve maternal and neonatal health.

## Introduction

Globally, about sixteen million girls aged 15 to 19 years and two million girls under the age of 15 give birth every year. Ninety five percent of these are in Sub Saharan countries [1]. Within individual countries in Sub Saharan Africa, adolescent births are more likely to occur among poor, less educated and rural populations because of reasons like; lack of access to contraception, early marriage, poor or limited access to education, cultural norms, poverty to mention but a few [2–4]. Adolescent pregnancy poses a challenge to improving maternal and child survival because pregnant adolescents and adolescent mothers are likely to be uneducated, unemployed and poor and might not therefore seek or utilize health care services for either themselves or their newborns’ at critical times [5–7]. Thus increased risks of maternal morbidity and mortality have been noted among pregnant and adolescents mothers [8].

Apart from the risk to themselves, babies born to adolescent mothers are also at risk of mortality and morbidity. The vulnerabilities of neonatal hood are compounded by the young maternal age thus increasing the risks for both maternal and child mortality. Among babies born to adolescents, higher risks of preterm births, low birth weight, stillbirths, and newborn deaths compared to babies born to older mothers have been previously reported [4,5,9–13]**.**

Uganda like other sub Saharan countries has high rates of teenage pregnancy. One quarter of adolescent girls have started reproduction, the neonatal mortality rate of adolescent born babies is at 43/1000 live births compared to 27/1000 live births among the babies of older women [14]. In this setting, community segregation and stigma attached to early pregnancy [15] coupled with the inability to afford financial costs of pregnancy, child birth and care [16] often times prevents the pregnant adolescents from seeking care, having the autonomy to make decisions [6] and accessing the best choices for themselves and their children’s health, resulting in critical delays and unnecessary deaths [17,18].

To reduce neonatal mortality and morbidity, the World Health Organization (WHO) recommends essential newborn care practices including promotion and support for early initiation of exclusive breastfeeding, thermal protection including promoting skin-to-skin contact, hygienic umbilical cord and skin care among others [19]. In Uganda, global strategies to reduce neonatal mortality like The Every Newborn Action Plan (ENAP) [20] have transcended into the Reproductive Maternal, Newborn and Child health Sharpened Plan for Uganda [21]. The Reproductive Maternal, Newborn and Child health Sharpened Plan for Uganda just like ENAP advocates for implementation of evidence based interventions to improve maternal and neonatal mortality focusing on vulnerable groups like adolescents to improve equity and access to health care for both the mother and newborn as a way of reducing mortality.

While evidence exists about essential newborn care practices among older women in Uganda [22–25], little has been documented among adolescent mothers.

This study therefore aimed to describe the essential newborn care practices and identify the associated factors among adolescent mothers in order to inform policy and development of feasible and sustainable community-based interventions that can improve the survival of newborn.

## Methods

### Study setting

This study was conducted in four sub counties in Hoima district. Hoima district is located in the mid-Western part of Uganda 200 km from Kampala, the capital city. Hoima has an estimated population of about 575,100 people and covers an area of 9442.9 sq. Km. Twenty three percent of adolescents in Hoima district have already started reproduction [14].

The district population is served by; 1 referral hospital, 2 Health Centre IV’s, 14 Health Centre III’s and 14 Health Centre II’s. These public facilities are complimented by 5 private dispensaries, 17 clinics and a team of 1023 community health workers, locally known as Village Health Team members (VHTs) offering home-based child health services spread out in the district.

### Study design, sampling method and data collection

This cross sectional study was conducted in the months of July and August 2014 using interviewer administered questionnaires. The required number of clusters and sample size was determined using Bennett’s method of cluster sampling [26]. Thus 82 clusters and a sample of 410 adolescent mothers was determined basing on the prevalence of dry cord care of 38% determined from a previous study in Eastern Uganda [25], a design effect of 1.036 [14] and a sampling error of 5%.

Lists of villages (clusters) from four randomly selected sub counties out of the seven that form Bugahya Health Sub District (HSD) were obtained from the planning department in Hoima district. Eighty two villages were then randomly selected using simple random sampling with replacement from a cumulative total of 208 villages that made up the four selected sub counties. In each selected village, VHTs or local council leaders guided research assistants to households with an adolescent mother having a child that was less than six months. Interviews were held with the consenting adolescent mothers in their homes. Where a household had two or more adolescent mothers, the youngest one was interviewed.

We recruited adolescent mothers aged 15 to 19 years who had children aged 1 to 6 months. Other studies done in Uganda have considered mothers with children of almost the same age group [25,27]. We excluded adolescent mothers who were sick or not at home at the time of the study.

Trained research assistants collected data using a questionnaire that had been previously pre tested and translated into Runyoro, the predominant local language in the area.

### Study outcome variables and data analysis

Three composite variables; appropriate neonatal breastfeeding, dry cord care and optimal thermal protection were generated from a list of seven recommended postpartum essential newborn care practices. Dry cord care was defined as no substance applied to the cord during the first seven days of life. Optimal thermal care was defined as a mother having practiced two or more optimal thermal protection practices (baby put skin-to-skin at birth or baby wrapped in warm clothes or the first bath delayed till twenty four or more hours). Appropriate neonatal breastfeeding was defined as initiating breastfeeding within the first one hour after birth and having practiced exclusive breastfeeding in the first six months. The above composite variables were dichotomized to Yes or No. Optimal thermal care and appropriate newborn feeding, the variables were dichotomized Yes if the mother reported any two or all the recommended practices under each category or No if 2 or more recommended practices were missing [25].

Maternal social demographics, child demographics, number of ANCs the adolescent mother attended, place of delivery, gender of the person that helped the adolescent mother in the postpartum period, skilled birth attendance, number of VHT visits during the neonatal period were assessed as some of the independent variables.

Data were entered using Epi info version 3.5.4, cleaned and later exported to STATA 12.0 for analysis. The svyset command in STATA was used to take care of the cluster effect of our data collection technique. Univariable analysis was done to describe the data while Odds ratio tests were used at bi-variable analysis to assess for any statistical associations between each dependent variable and each of the independent variables. All statistical tests were considered significant at p <0.05. At multivariate analysis, all variables with a p value less than 0.2 together with those previously cited in literature to have a bearing on essential newborn care were fitted in logistic regression models to identify factors independently associated with each outcome. A variable was considered statistically significant if the p <0.05.

### Ethics

The Makerere University School of Public Health Higher Degrees research and Ethics committee and Hoima listrict local government respectively approved the study protocol, the consent procedure and study tools. In this study, per ethics approval, adolescent mothers below 18 years were considered to be emancipated minors, so written informed consent was obtained from each adolescent mother before the interviews began. Each adolescent mother who consented to take part in the study was thus required to append a signature or a thumb print to the consent form.

## Results

All the eligible adolescent mothers approached consented to participate in the study thus a total of 410 adolescent mothers were interviewed. The mean age was 17.9 (SD ± 1.11). Fifty five percent (227/410) were married or co habiting, 64.3% (264/410) had obtained primary education and only 27.1% (111/410) were currently employed. Only 52.4% (215/410) had attended the recommended four ANCs before delivery. Seventy eight percent (323/410) had delivered in a health facility, and 52.0% (113/217) had received three or more postnatal home visits by VHTs (Table 1).

**Table 1:** Background characteristics of adolescent mothers.

Characteristic	Frequency	(%)
Age (n = 410)		
15	7	1.7
16	51	12.4
17	68	16.6
18	107	26.1
19	177	43.2
Parity (n = 410)		
1	295	72.0
2	93	22.7
3	18	4.4
4	4	1.0
Marital status (n = 410)		
Not married/ single	183	44.6
Married/ cohabiting	227	55.4
Highest level of education (n = 410)		
None	40	9.8
Primary	264	64.4
Secondary and beyond	106	25.8
Occupation (n = 410)		
Un employed	299	72.9
Employed	111	27.1
Number of ANC attended (n = 410)		
None	6	1.5
1	22	5.4
2	61	14.9
3	106	25.8
4+	215	52.4
Place of delivery(n = 410)		
Health facility	323	78.8
Outside health facility	87	21.2
Type of Delivery (n = 410)		
Vaginal	389	95.6
Caesarean section	18	4.4
Number of VHT visits in the postnatal period (n = 217)		
1	52	24.0
2	52	24.0
3	113	52.0

### Newborn care practices

Only 31% (127/410) adolescent mothers practiced dry cord care. Majority of the newborns’ had potentially hazardous substances applied to the cord. Fifty one percent (146/283) had applied salty water, 20.1% (57/283) local herbs and 11.7% (33/283) applied talcum powder (Table 2).

**Table 2:** Essential Newborn Care practices by the adolescent mother.

ENC practice	Frequency	Percentage (%)
**CORD CARE PRACTICES**		
Dry cord care (n = 410)	127	31
**Substances applied to cord (n = 283)**		
Salty water	146	51.6
Local herbs	57	20.1
Powder	33	11.7
Soot	29	10.2
Surgical spirit	10	3.5
Animal droppings	3	1.1
Others	5	1.8
**THERMAL CARE PRACTICES**		
**Optimal thermal care** (n = 410)	276	67.3
Skin to skin care (n = 117)	81	69.2
Baby wrapped in warm clothes	349	85.1
First bath delayed by 24 hrs	272	66.3
**NEWBORN FEEDING PRACTICES**		
Appropriate breastfeeding practice (n = 410)	248	60.5
Initiated of breastfeeding within 1 hr	291	71.0
**First feed given to newborn**		
Breast milk	318	77.6
Water	42	10.2
Glucose	44	10.7
Infant formula	4	1.0
Others	2	0.5

Sixty seven percent (276/410) were judged to have practiced the recommended thermal protection. As per the individual thermal care practices, 85.1% (349/410) reported that they had wrapped their babies in warm clothes, 66.3% (272/410) had delayed their newborns’ first bath by twenty four hours.

Appropriate newborn feeding was practiced by 60.5% (248/410) of the adolescent mothers. Initiation of breast feeding within one hour and colostrum being given were nearly universal in this study group at 71% (291/410) and 89.8% (368/410) respectively (Table 2).

### Factors associated with essential newborn care practices among adolescent mothers

#### Dry cord care

At bi-variate analysis, adolescent mothers who had knowledge of dry cord care (crude odds ratio 4.39, 95% CI (2.28–8.07) and a male caretaker in the postnatal period (crude odds ratio 2.09, 95% CI (1.11–3.97) were more likely to report dry cord care while those that had delivered twins (crude odds ratio 0.28, 95% CI (0.10–0.78) were less likely to report having practiced dry cord care. After logistic regression, we found the independent predictors of dry cord care to be knowledge of dry cord care (Adjusted odds ratio 5.34, 95% CI (1.51–18.84) and having delivered twins (adjusted odds ratio 0.04, 95% CI (0.01–0.22) (Table 3).

**Table 3:** Factors associated with essential newborn care practices among adolescent mothers in Hoima District.

	Dry cord care				Optimal thermal care				Appropriate breast feeding			
VARRIABLE	Crude odds ratio(CI)	P_Value	Adjusted odds ratio (CI)	P_Value	Crude odds ratio(CI)	P_Value	Adjusted odds ratio (CI)	P_Value	Crude odds ratio(CI)	P_Value	Adjusted odds ratio (CI)	P_Value
Child sex												
Male	1		1									
Female	1.65(1.09–2.49)	0.017*	1.71(0.88–3.31)	0.113								
Child birth weight												
More than2.5 Kgs					1		1					
Less or equal to 2.5kg					1.76(1.02–3.03)	0.041	1.34(0.48–3.73)	0.565				
Number of children at birth												
Single	1		1									
Twin	0.28(0.10–0.78)	0.015	0.04(0.01–0.22)	0.000**								
Number of ANC attended												
1_3	1		1		1		1		1		1	
4 or more	1.30(1.08–1.56)	0.006*	2.17(0.90–5.21)	0.083	1.20(1.03–1.40)	0.018	0.96(0.32–2.92)	0.945	1.00 (0.87–1.14)	0.948	1.08(0.58–2.08)	0.261
Facility delivery												
No	1		1		1		1		1			
Yes	1.54(0.86–2.75)	0.148	1.21(0.30–4.95)	0.787	2.34(1.32–4.18)	0.004	3.26(0.28–38.47)	0.342	1.32(0.75–2.32)	0.329	0.57(0.42–1.84)	0.719
Type of delivery												
Normal									1		1	
Caesarian									0.12 (0.03–0.46)*	0.002	0.19(0.04–0.96)	0.044**
Duration of hospital stay postpartum												
Went home within a day	1		1		1		1		1		1	
Spent more than one day	1.69(0.81–3.51)	0.159	1.04(0.29–3.74)	0.948	1.35(0.75–2.42)	0.311	1.50(0.40–5.62)	0.541	3.69 (2.10–6.49)*	0.000*	2.45(1.23–4.86)	0.011**
Number of VHT postnatal visits												
1 visit	1		1		1		1					
3 or more visits	1.43(1.00–2.04)	0.051	2.33(0.92–5.88)	0.073	1.29(0.91–1.83)	0.15	1.25(0.51–3.04)	0.945				
Gender of caretaker in the postnatal period												
Female	1		1						1		1	
Male	2.10(1.11–3.97)	0.024*	1.94(0.17–22.22)	0.588					0.55 (0.25–1.23)	0.142*	0.18(0.07–0.49)	0.001**
Caretakers education level												
Primary and below					1		1		1		1	
Secondary and above					1.88(0.96–3.56)	0.064	1.10(0.34–3.57)	0.869	0.82 (0.45–1.51)	0.524	0.86(0.36–2.05)	0.722
Knowledge of essential newborn care practice												
No	1		1		1		1		1		1	
Yes	4.39(2.28–8.07)	0.000*	5.34(1.51–18.84)	0.000*	15.91(8.72–29.02)	0	25.15(7.01–90.20)	0.000**	15.51(6.06–39.69)	0.000*	14.71(5.20–41.58)	0.000**

*significant at bivariate analysis.

** significant at multivariate analysis

#### Optimal thermal protection

Adolescent mothers who had attended four or more ANC (Crude odds ratio 1.20, 95% CI (1.03–1.40), those knowledgeable about thermal care practices (crude odds ratio 15.91, 95% CI (8.72–29.02) and those that had delivered low birth weight babies (Crude odds ratio 1.76, 95% CI (1.02–3.03) were more likely to report having practiced the recommended optimal thermal protection. While the adolescent mothers who had delivered outside health facilities (Crude odds ratio 0.43, 95% CI (0.24–0.76) were less likely to report practice of optimal thermal protection as compared to those who had delivered in health facilities. Surprisingly, knowledge (Adjusted odds ratio 25.15, 95% CI (7.01–90.20) was the only independent predictor of optimal thermal care (Table 3).

#### Optimal neonatal breastfeeding

At bi-variate analysis, having had a caesarean birth (crude odds ratio 0.12, 95% CI (0.03–0.46), a male caretakers in the postnatal period (crude odds ratio 0.55, 95% CI (0.25–1.23) were negatively associated with the practice of the recommended newborn feeding while having stayed in hospital for more than a day postpartum (crude odds ratio 3.69, 95% CI (2.10–6.49) and knowledge of the correct time to initiate breastfeeding (crude odds ratio 15.51, 95% CI (6.06–39.69) were the positive predictor for practice of appropriate neonatal feeding.

In the adjusted analysis, the independent predictors of optimal newborn breast feeding were: staying in hospital for more than a day postpartum (adjusted odds ratio 2.45, 95% CI (1.23–4.86), knowledge (adjusted odds ratio 14.71, 95% CI (5.20–41.58), having had a caesarean birth (adjusted odds ratio 0.19, 95% CI (0.04–0.96), and a male caretakers in the immediate postnatal period (adjusted odds ratio 0.18, 95% CI (0.07–0.49) (Table 3).

## Discussion

In this study, we found sub optimal practices in relation to cord care, thermal protection and breastfeeding among adolescent mothers. Care for newborns’ was characterised by applying potential harmful substances to the cord stump, early bathing and giving pre- lacteal feeds. These findings are not very different from what previous studies that have reported about essential newborn care practices among women of the general reproductive age in rural Uganda [22–25]. A qualitative study found that adolescent mothers learnt how to care for their newborns by observing what older mothers did [28]. However, older mothers may not always exhibit recommended newborn practices (27).

### Cord care practices

Adolescent mothers exhibited poor cord care practices. Seven out of ten adolescent mothers had placed potentially harmful substances like salty water, local herbs, talcum powder on the newborns cords. Similar findings have been previously reported in other settings among adolescent mothers [29] and among women of the general reproductive age group in Nepal [30] and in Uganda [27]. Reasons such as the perceived fast healing, quicker falling off of the cord stump and traditional beliefs have been elucidated previously for poor cord care [23,25,31]. Applying substances on the cord exposes the newborns to infections like sepsis**.** Infections are one of the leading causes of neonatal mortality in Sub Saharan Africa [17] and in Uganda [32].

Interestingly, although 62% were found knowledgeable of dry cord care, only 31% reported having practiced dry cord care. This is indicative of a knowledge to practice gap. We hypothesize this could be explained by two reasons; 1) the adolescent mothers learn or are influenced by older women who might not always be the source of correct information and 2) the belief that applying substances could help facilitate fast healing. Previously, Waiswa et al [23] and Mangwi et al [28] have elucidated similar reasons to explain poor cord care.

We also found that adolescent mothers who had delivered twins were found less likely to practice dry cord care. In the study area, having a multiple birth or being a twin is associated with a lot of cultural and traditional practices associated with smearing herbs on twin babies bodies. Often these cultural practices might expose the children to potentially harmful practices in the neonatal period ranging from deprived feeding to additives or substances being placed on the cord stump. Although the World Health Organisation recommends dry cord care, it gives provision for use of chlorhexidine in situations only to replace application of a harmful traditional substance, such as cow dung to the cord stump [33]. In Uganda, dry cord care is still the recommended practice for cord care.

### Optimal thermal care practices

Adolescent mothers who had no knowledge of the recommended optimal thermal protection practices were found less likely to have practiced optimal thermal protection. Sub optimal thermal protection practices characterised by early bathing within 24 hours of delivery and minimal practice of skin to skin care were reported. Previously, Mangwi et al and Byaruhanga et al have reported sub optimal thermal care among women of the general reproductive age [27,34]. Among adolescent mothers and some health workers, poor knowledge and poor acceptance of simple methods to maintain a warm chain from birth till the end of the neonatal period have been documented as risk factors for poor neonatal thermal care [25,27,31,34,35].

### Breastfeeding practices

Sixty percent of the adolescent mothers had initiated breastfeeding within one hour after birth. This is different from findings in India where less than fifty percent of adolescent mothers reported initiation of breastfeeding within one hour [29]. In this study, staying for more than one day in hospital postpartum was associated with the practice of appropriate newborn feeding. This finding could be explained by the fact that adolescent mothers who had stayed longer in hospital were more likely to benefit from the knowledge and supervised postnatal care by health workers for both the mother and newborn [14]. According to the Sexual and Reproductive Health Guidelines of Uganda, a newborn is expected to receive a postnatal check-up (including ensuring initiation of breastfeeding) within the first 24 hours of life [36]. In areas of high neonatal mortality, the timing, package and provider of postnatal care play a critical role in initiating mothers and caregivers into the recommended essential newborn practices [33,37]. Having had a male caretaker was a risk factor for poor newborn feeding practices. In this setting, traditionally the caretaking role is mainly by women, so men are unlikely to know when to initiate or how to feed the newborn and may not be in position to guide the adolescent mothers about appropriate newborn feeding practices. In Uganda, there has been low male involvement in activities like ANC and postnatal care which are critical for learning about essential newborn care practices. This may explain the low levels of knowledge of essential newborn care among men [38], hence inability to mentor the adolescent mothers**.** Although no literature was found to corroborate any association between male caregivers and breastfeeding practices in an African setting, a comparative cross sectional study done in Bangladesh noted that although men generally had low knowledge levels of newborn care, it could be enhanced through behaviour change communication [39] and involving men in ANC activities.

#### Methodological considerations

One key limitation of this study could have been recall bias. The study was not able to observe the newborn care practices but rather depended solely on recall and maternal accounts. To minimize recall bias, we limited our interviews to only mothers with babies up to six months.

Secondly, not all villages had registers of recently delivered mothers. As a result the VHTs in those villages were enlisted to record the adolescent mothers before the study started. This could have resulted in incomplete VHT lists but also inability to rule out the probability that the VHT might have recorded only those adolescent mothers that they had visited.

## Conclusion

Findings revealed sub optimal essential newborn care practices among adolescent mothers. Applying potentially harmful products on the cord stump, sub optimal thermal care and early bathing were the major indicators of poor cord care and optimal thermal protection practices among adolescent mothers.

Adolescent mothers should be a focus of strategies to improve maternal and neonatal health. Innovative strategies like special ANC days for pregnant adolescents and a follow up mechanism after delivery at community level by community health workers could improve essential newborn care among adolescents.

## Supporting Information

S1 File Adolescent Mother Questionnaire. (DOCX)

S2 File Raw data Essential Newborn care among adolescent mothers. (XLSX)

## References

[1] WHO (2011) WHO guidelines on preventing early pregnancy and poor reproductive outcomes among adolescents in developing countries. 10.1016/j.jadohealth.2013.03.002 23608717

[2] Vogel JP, Pileggi-Castro C, Chandra-Mouli V, Pileggi VN, Souza Jã P, et al (2015) Millennium Development Goal 5 and adolescents: looking back, moving forward. Arch Dis Child 100: S43–47. 10.1136/archdischild-2013-305514 25613967 PMC4316852

[3] Kurth F, Bélard S, Mombo-Ngoma G, Schuster K, Adegnika AA, et al (2010) Adolescence as risk factor for adverse pregnancy outcome in Central Africa–a cross-sectional study. PLoS One 5: e14367 10.1371/journal.pone.0014367 21188301 PMC3004789

[4] Ganchimeg T, Ota E, Morisaki N, Laopaiboon M, Lumbiganon P, et al (2014) Pregnancy and childbirth outcomes among adolescent mothers: a World Health Organization multicountry study. BJOG: An International Journal of Obstetrics & Gynaecology 121: 40–48. 10.1111/1471-0528.12630 24641534

[5] Uzun AK, Orhon FS, Baskan S, Ulukol B (2013) A comparison between adolescent mothers and adult mothers in terms of maternal and infant outcomes at follow-ups. J Matern Fetal Neonatal Med 26: 454–458. 10.3109/14767058.2012.733748 23020604

[6] Singh L, Rai RK, Singh PK (2012) Assessing the utilization of maternal and child health care among married adolescent women: evidence from India. Journal of biosocial science 44: 1 10.1017/S0021932011000472 21933465

[7] Rahman MM, Haque SE, Zahan MS (2011) Factors affecting the utilisation of postpartum care among young mothers in Bangladesh. Health Soc Care Community 19: 138–147. 10.1111/j.1365-2524.2010.00953.x 20880103

[8] WHO (2012) Early marriages, adolescent and young pregnacies. Geneva.

[9] Hidalgo LA, Chedraui PA, Chávez MJ (2005) Obstetrical and neonatal outcome in young adolescents of low socio-economic status: a case control study. Archives of gynecology and obstetrics 271: 207–211. 10.1007/s00404-004-0600-7 15029506

[10] Kongnyuy EJ, Nana PN, Fomulu N, Wiysonge SC, Kouam L, et al (2008) Adverse perinatal outcomes of adolescent pregnancies in Cameroon. Maternal and Child health journal 12: 149–154. 10.1007/s10995-007-0235-y 17562148

[11] Okigbo CC, Speizer IS (2015) Determinants of Sexual Activity and Pregnancy among Unmarried Young Women in Urban Kenya: A Cross-Sectional Study. PLoS One 10: e0129286 10.1371/journal.pone.0129286 26047505 PMC4457813

[12] Nankabirwa V, Tumwine JK, Tylleskär T, Nankunda J, Sommerfelt H, et al (2011) Perinatal Mortality in Eastern Uganda: A Community Based Prospective Cohort Study. PLoS One 6: e19674 10.1371/journal.pone.0019674 21573019 PMC3090412

[13] Jutte DP, Roos NP, Brownell MD, Briggs G, MacWilliam L, et al (2010) The Ripples of Adolescent Motherhood: Social, Educational, and Medical Outcomes for Children of Teen and Prior Teen Mothers. Academic Pediatrics 10: 293–301. 10.1016/j.acap.2010.06.008 20674531

[14] UBOS I (2012) Uganda Demographic and Health Survey 2011 Kampala Uganda: UBOS and Calverton, Maryland: ICF International Inc.

[15] Atuyambe L, Mirembe F, Tumwesigye NM, Annika J, Kirumira EK, et al (2008) Adolescent and adult first time mothers' health seeking practices during pregnancy and early motherhood in Wakiso district, central Uganda. Reproductive Health 5: 13 10.1186/1742-4755-5-13 19116011 PMC2648938

[16] Ganchimeg T, Ota E, Morisaki N, Laopaiboon M, Lumbiganon P, et al (2014) Pregnancy and childbirth outcomes among adolescent mothers: a World Health Organization multicountry study. BJOG: An International Journal of Obstetrics & Gynaecology 121: 40–48. 10.1111/1471-0528.12630 24641534

[17] Kinney MV, Kerber KJ, Black RE, Cohen B, Nkrumah F, et al (2010) Sub-Saharan Africa's mothers, newborns, and children: where and why do they die? PLoS medicine 7: e1000294 10.1371/journal.pmed.1000294 20574524 PMC2888581

[18] Atuyambe L, Mirembe F, Tumwesigye N, Annika J, Kirumira E, et al (2008) Adolescent and adult first time mothers' health seeking practices during pregnancy and early motherhood in Wakiso district, central Uganda. Reproductive Health 5: 13 10.1186/1742-4755-5-13 19116011 PMC2648938

[19] WHO (1996) Essential newborn care.

[20] WHO (2014) Every Newborn: an action plan to end preventable deaths.

[21] MOH (2013) Reproductive Maternal,Newborn and Childhealth sharpened plan for Uganda. Kampala: MOH.

[22] Waiswa P, Kallander K, Peterson S, Tomson G, Pariyo GW (2010) Using the three delays model to understand why newborn babies die in eastern Uganda. Tropical Medicine & International Health 15: 964–972. 20636527 10.1111/j.1365-3156.2010.02557.x

[23] Waiswa P, Kemigisa M, Kiguli J, Naikoba S, Pariyo GW, et al (2008) Acceptability of evidence-based neonatal care practices in rural Uganda–implications for programming. BMC pregnancy and childbirth 8: 21 10.1186/1471-2393-8-21 18570672 PMC2459143

[24] Byaruhanga RN, Nsungwa-Sabiiti J, Kiguli J, Balyeku A, Nsabagasani X, et al (2011) Hurdles and opportunities for newborn care in rural Uganda. Midwifery 27: 775–780. 10.1016/j.midw.2010.02.005 20685016

[25] Waiswa P, Peterson S, Tomson G, Pariyo GW (2010) Poor newborn care practices—a population based survey in eastern Uganda. BMC Pregnancy Childbirth 10: 9 10.1186/1471-2393-10-9 20178626 PMC2834614

[26] Bennett S, Woods T, Liyanage WM, Smith DL (1991) A simplified general method for cluster-sample surveys of health in developing countries. World Health Stat Q 44: 98–106. 1949887

[27] Mangwi Ayiasi R, Kasasa S, Criel B, Garimoi Orach C, Kolsteren P (2014) Is antenatal care preparing mothers to care for their newborns? A community-based cross-sectional study among lactating women in Masindi, Uganda. BMC pregnancy and childbirth 14: 114 10.1186/1471-2393-14-114 24667001 PMC3987096

[28] Ayiasi M, Van Royen K, Verstraeten R, Atuyambe L, Criel B, et al (2013) Exploring the focus of prenatal information offered to pregnant mothers regarding newborn care in rural Uganda. BMC pregnancy and childbirth 13: 176 10.1186/1471-2393-13-176 24041135 PMC3848633

[29] Rahman M, Haque SE, Zahan S, Islam O (2011) Noninstitutional births and newborn care practices among adolescent mothers in Bangladesh. J Obstet Gynecol Neonatal Nurs 40: 262–273. 10.1111/j.1552-6909.2011.01240.x 21585526

[30] Sreeramareddy CT, Joshi HS, Sreekumaran BV, Giri S, Chuni N (2006) Home delivery and newborn care practices among urban women in western Nepal: a questionnaire survey. BMC pregnancy and childbirth 6: 27 10.1186/1471-2393-6-27 16928269 PMC1560161

[31] Karas DJ, Mullany LC, Katz J, Khatry SK, LeClerq SC, et al (2012) Home Care Practices for Newborns in Rural Southern Nepal During the First 2 weeks of Life. Journal of tropical pediatrics 58: 200–207. 10.1093/tropej/fmr057 21705765 PMC3530276

[32] Mbonye AK, Sentongo M, Mukasa GK, Byaruhanga R, Sentumbwe-Mugisa O, et al (2012) Newborn survival in Uganda: a decade of change and future implications. Health Policy Plan 27 Suppl 3: iii104–117. 22692413 10.1093/heapol/czs045

[33] WHO (2013) WHO recommenations on post natal care for the mother and newborn.

[34] Byaruhanga R, Bergstrom A, Okong P (2005) Neonatal hypothermia in Uganda: prevalence and risk factors. Journal of tropical pediatrics 51: 212–215. 10.1093/tropej/fmh098 15917265

[35] Shamba D, Schellenberg J, Hildon ZJ-L, Mashasi I, Penfold S, et al (2014) Thermal care for newborn babies in rural southern Tanzania: a mixed-method study of barriers, facilitators and potential for behaviour change. BMC pregnancy and childbirth 14: 267 10.1186/1471-2393-14-267 25110173 PMC4141124

[36] MOH (2011) The National Policy Guidelines and Service Standards for Sexual and Reproductive Health and Rights In: Reproductive Health Division DoCH, editor. Three ed Kampala: Ministry of health.

[37] Sines E, Syed U, Wall S, Worley H (2007) Postnatal care: A critical opportunity to save mothers and newborns. Policy Perspectives on Newborn Health.

[38] Tweheyo R, Konde-Lule J, Tumwesigye NM, Sekandi JN (2010) Male partner attendance of skilled antenatal care in peri-urban Gulu district, Northern Uganda. BMC pregnancy and childbirth 10: 1–9. 10.1186/1471-2393-10-53 20846369 PMC2946269

[39] Nasreen HE, Leppard M, Al Mamun M, Billah M, Mistry SK, et al (2012) Men’s knowledge and awareness of maternal, neonatal and child health care in rural Bangladesh: a comparative cross sectional study. Reproductive Health 9: 18 10.1186/1742-4755-9-18 22943448 PMC3453489

