# Supplementary material for: Newborn Care Practices among Adolescent Mothers in Hoima District, Western Uganda
Source: PLoS One. 2016 Nov 17;11(11):e0166405. doi: 10.1371/journal.pone.0166405 (PMC5113955; doi:10.1371/journal.pone.0166405)
Supplement: S1 File — (DOCX) [file pone.0166405.s001.docx]

**ADOLESCENT MOTHER QUESTIONNAIRE**

**STUDY TITLE: An assessment of essential newborn care practices among adolescent mothers in Hoima District**

| Record Number: |  |  |  |
| --- | --- | --- | --- |

| Cluster number: |  |  |  |
| --- | --- | --- | --- |

| Interviewer number |  |  |
| --- | --- | --- |

| Date of Interview |  |  |  |
| --- | --- | --- | --- |

**Section 1: Socio-demographic Characteristics of adolescent mother. (***For questions 3 to 9, please circle only one answer)*

| 1 | Age of respondent in complete years |  |  |
| --- | --- | --- | --- |
| 2 | Date of birth |  |  |
| 3 | Parity | 1  2  3  4+ |  |
| 4 | Respondents religion | Anglican………………………..…1  Catholic……………………….…..2  Adventist…………………….……3  Muslim………………………….…4  Pentecostal…………………..…….5 |  |
| 5 | Marital status | Single………………………………1  Married/ Cohabiting……………….2  Divorced /separated……………….3  Widow ……………………….…….4 |  |
| 6 | Highest level of education of mother | None……………………….……….1  Primary…………………………...2  O level………………………...…….3  A level………………………..…….4  Tertiary…………………………….5 |  |
| 7 | What is your tribe | Munyoro …………………………..1  Mukiiga ……………..……………..2  Alur……………………..………….3  Lugbara ………………….………..4  Muganda ………………………….5  Others specify--------------------- |  |
| 8 | Occupation | Fulltime house wife………..…….…1  Business …………….....................2  Private sector.………..…………….3  None……………………..…………4  Others specify-------------------- |  |
| 9 | Where do you currently stay? | At my parents home……………….1  My in laws home…………………...2  My husband home…………………3  Others specify………………………4 |  |

**Section 2; Now I am going to ask you questions about (baby’s name). (***Please circle only one answer)*

| 10 | How old is (baby’s name) now?  **Please record age in months** |  |  |
| --- | --- | --- | --- |
| 11 | When was baby born? |  |  |
| 12. | Baby’s sex | Male………………………………1  Female……………….……………2 |  |
| 13 | Is (baby’s name) a singleton birth? | Yes ……………………….……….. 1  No ………………………………...2 | If yes, skip to 14 |
| 14 | Are they all surviving? | Yes ……………………………….. 1  No ………………………………...2 |  |
| 15 | At how many months did you deliver (baby’s name)? | Nine………………….………..........1  Eight…………………….………....2  seven………………………………3 |  |
| 16a | How big was (baby’s name) at birth? | ≥ 2500gms…………………..……1  ≤ 2500gms…………………….....2 | *Pick answer from child health card/passport* |
| 16 b | How big was (baby’s name) at birth? | Big……………………….………...1  Average……………………………2  Small………………………………3 | *Skip if mother has a child card* |

**SECTION 3; ANC, delivery practices. (***For questions 17 -27, circle only one answer***).**

| 17 | Did you attend ANC when pregnant with baby? | Yes ………………………….. 1  No …………………………...2 | ***If no, skip to Q.19*** |
| --- | --- | --- | --- |
| 18 | If yes, from where? | Gov’t/public……..…………..1  PFP….……………………......2  PNFP…..……………….……..3 |  |
| 19 | How many times before delivery? | Once...…………………….….1  Twice…………………………2  Thrice………………………3  Four times………………….4  Four or more times………………5 |  |
| 20 | Where did you deliver this baby from? | Gov’t facility…………...……….1  Maternity home………..……….2  PNFP…………………………….3  Outside facility…………………4 | If 1-3 go to Q.21 |
| 21 | **For outside facility deliveries only**, Did you have a mama kit? | Yes ………………..……….. 1  No ……………………..…...2 |  |
| 22 | How did you deliver? | Normally ………………..….1  Caesarian section……………..2 |  |
| 23 | How many days did you stay in hospital? | Went home that day………….1  One day……………………….2  2 to 4 days………………….....3  5 to 10 days…………………..4  Others specify………………………..5 |  |
| 24 | Is there a VHT in your Village? | Yes ………………………….. 1  No ……………….…………...2 | ***If no, Skip to Q.28*** |
| 25 | Did the VHT visit you when you produced (name of baby)? | Yes ………………….……….. 1  No …………………….……...2 |  |
| 26 | If yes how many times? | Once………………………..…1  Twice……………..…………...2  Thrice……………….…………3  3+ times………………………4  None…………………….…….5 |  |
| 27 | Did the VHT tell you anything about how to take care of the baby? | Yes …………………….…….. 1  No ………………………..…...2 | ***If no Skip to Q.28*** |
| 28 | If yes, what did she/ he talk about?  **(Circle all mentioned answers)** | How to breastfeed……………..1  To keep baby warm…….………2  To take baby for immunization……………….…..3  To sleep under a mosquito net…………………….…………4  Others specify……………… |  |

**SECTION 4: Immediate postnatal care.** *(For questions 29-34, please circle only one answer***)**

| 29 | Within 7 days of delivery did anybody: | \| Assess the temperature for (baby’s name) \| Y  1 \| N  2 \| \| --- \| --- \| --- \| \| Counsel you on breastfeeding \| 1 \| 2 \| \| Observe your baby breastfeeding? \| 1 \| 2 \| \| Weigh the baby \| 1 \| 2 \| \| Examine (baby’s name) cord? \| 1 \| 2 \| \| Counsel you on danger signs for newborns? \| 1 \| 2 \| |  |
| --- | --- | --- | --- | --- | --- | --- | --- | --- | --- | --- | --- | --- | --- | --- | --- | --- | --- | --- | --- | --- | --- |
| 30 | Who took care of you immediately after delivery of (baby’s name)? | My sister…………………………………….....1  My mother……………........................................2  My mother in law.……………………….……..3  My sister in law……………..…..........................4  My husband…………………….,……………5  Myself.……………………………………..6 |  |
| 31 | What is their highest level of education? | None……………………………………………1  Primary…………………………………………2  O Level …………………….………..………….3  A Level……………..///……………………….4  Tertiary…………………..………….…………5 |  |
| 32 | **For women caretakers only**. Does she have children? | Yes …………………………………………….. 1  No ………………………………………….…...2 |  |
| 33 | **If yes,** how many children? | 1  2  3  4  5+ |  |
| 34 | **For any care giver,** did they give you advice on how to take care of (baby’s name) for | \| Breast feed \| Y  1 \| N  2 \| \| --- \| --- \| --- \| \| Optimal thermal protection \| 1 \| 2 \| \| Cord care \| 1 \| 2 \| \| Danger signs \| 1 \| 2 \| |  |

**SECTION 5: Cord care. (***For questions 35, 36 and 38 circle only one answer. Circle all mentioned answers for question 37)*

| **35** | How is the cord of the baby supposed to be cared for? | Dry cord care/ do not Apply anything…………………………...1  Others  Specify………………….…………2 |  |
| --- | --- | --- | --- |
| **36** | At home, did you put anything on the umbilical cord before it fell off? | Yes ……………………………….. 1  No ………………………………...2 |  |
| **37** | If yes, what did you apply? | Surgical Spirit.………..……………1  Salty water………………………...2  Animal droppings…………………3  Soil………………….……………..4  Soot……………………….……….5  Local herbs……………..………..6  Others………………………………………………………………….….7 |  |
| **38** | Who advised you about that? | Health worker..…………......1  Relative .………....................2  VHT…………...……………3  Other specify………………………4 |  |

**SECTION 6: Thermal protection. (***For questions 39-40, circle all mentioned answers. Question 41 - 42 circle only one answer).*

| 39 | How is a newborn supposed to be kept warm? | Contact with mothers skin...........................1  Wrapping in clothes......................................2  Others specify...............................................3 |  |
| --- | --- | --- | --- |
| 40 | What did you do to warm (baby’s name) after delivery? | Clothed baby head to toe…….....…..........1  Skin to skin...…………………....................2  Kept baby close to me immediately after birth………...................................................3 |  |
| 41 | After how long is a baby supposed to be bathed? | After 24 hours...............................................1  Immediately after birth.................................2  Others specify..............................................3 |  |
| 42 | How long after delivery was (baby’s name) bathed? | Within24hrs…………………………….1  After 24 hrs……………………................2 |  |

**SECTION 7: Breastfeeding. (***Apart from question 48, circle only one answer for the other questions)*

| 43 | When is a newborn supposed to be breast fed? | Within 1 hr of birth/ immediately after birth.................................................1  Others specify...................................2 |  |
| --- | --- | --- | --- |
| 44 | What was the first feed you gave (baby’s name) | Breast fed…………………...……1  Formula……………………....…..2  Water…………………………..…3  Glucose………...…………………4  Others specify...................................5 |  |
| 45 | Approximately how long after delivery did you first breastfeed your baby? | Within 30 minutes……….....……..1  30 minutes to 1 hr……………...….2  2 hrs…………………………….....3  6 hrs…………….....………………4  24 hrs……………….....…………..5  After 24 hrs.......................................6 |  |
| 46 | Did you give colostrum? | Yes …………………………..…….. 1  No ……………………………….....2 | If yes skip to Q 48 |
| 47 | **If no,** Why did not you give colostrum | Looked dirty…………….……….…..1  Taboo………………………………...2  Advised against it by caretaker ….…3 |  |
| 48 | **If after baby was breast fed after 24 hrs,** why did you take that long to breastfeed? | No breast milk……………….........….1  Did not know how to breast feed…………………………......…...2  Was giving other feeds……....…..….3  Baby wasn’t crying……….......…….4  Do not know……………….......…….5 |  |
| 49 | **If baby’s first feed was not breast milk,** why did you give that other feed? | No breast milk………….....……….1  Was advised to…………....……….2 |  |
| 50 | If advised, who advised you | Health worker…………......……..…1  Caretaker………………….....….…2 |  |
| 51 | Did you exclusively breastfeed (baby’s name) throughout the first month? | Yes ……………………………….... 1  No ……………………………..........2 |  |
| 52 | How often did do you breast feed (baby’s name) in a day? | 4 times…………….......……………1  8-10 times……………......………….2  Whenever cried…………......………3 |  |

**SECTION 8: Danger signs**

| 53 | Do you know about some signs that can alert you that (baby’s name) is sick or not ok? | No ………………………………..0  Yes …………………………..…...1 | If no skip to Q.54 |
| --- | --- | --- | --- |

54. What do you consider to be a newborn danger signs in a newborn (circle all mentioned)

| Danger sign |  |
| --- | --- |
| Very small baby | 1 |
| Baby crying too much | 2 |
| Un able to breast feed | 3 |
| Yellowing of the baby | 4 |
| Baby is too hot | 5 |
| Baby is too cold | 6 |
| Poor cry from baby | 7 |
| Too sleepy difficult to wake | 8 |
| Convulsion or fit is | 9 |
| Smelly /reddish umbilical cord | 10 |
| Skin pustules | 11 |
| Red, swollen eyelids, and pus discharge from eyes | 12 |
| Baby is grunting | 13 |

| 55 | Who assists you financially in caring for (baby’s name) | Father of baby……………………1  My parents………………………..2  My siblings……………………….3  My in laws……………………….4  Self….……………………………5  Others specify…………………….6 |  |
| --- | --- | --- | --- |

Any comments /questions?

Thank you for your time

**TRANSLATED ADOLESCENT MOTHER QUESTIONNAIRE**

**Endolera yenkerembe omu abaiski be myaka 15-19 omu nyamasaaza ya Hoima**

| Record Number: |  |  |  |
| --- | --- | --- | --- |

| Cluster number: |  |  |  |
| --- | --- | --- | --- |

| Interview number |  |  |  |  |
| --- | --- | --- | --- | --- |

| Date of Interview |  |  |  |
| --- | --- | --- | --- |

**Ekichweka kyokubanza: Ebikwatana ha muzaire wa omwana (Byona bikuru. 1-2, handikamu. 3-8 taho e manyikirizo ha sura emu buli kikaguzo. Ebikaguzo byona biina kwijulizibwa)**

| 1 | Oina emyaka e’ingaha |  |  |
| --- | --- | --- | --- |
| 2 | Okazarwa myaka ki? |  |  |
| 3 | Oina amazaro a’ingaha | 1  2  3  4+ |  |
| 4 | Osoma diini ki? | kuristayo………………………….…1  katoliki…………………………..…..2  Adiventi………………………..……3  Musiramu……………………………4  Murokole…………………………….5 |  |
| 5 | Ebyobuswezi | Tinka s’werwaga……………………..1  Ndi mufumbo/ Nyikara no musaijja ………………………………………..2  Mufakati.……………………………..3 |  |
| 6 | Ebyensoma yawe | Ntasoome…………………………….1  Purimare……………………………...2  O’ level ………………………………3  Haaya ………………………………..4  Insituti………………………………..5 |  |
| 7 | Oli wa kabila ki? | Munyoro ………………………….…1  Mukiiga ……………………………..2  Alur………………………………….3  Lugbara ……………………………..4  Muganda …………………………….5  Ebindi**---------------------** |  |
| 8 | Entahya yawe | Nyikara muka…..…………….………1  Nkoo’ra abandi……………….............2  Business………………………………3  Tinyina……………………………….4 |  |
| 9 | Kuruga wa Zaire oikara nkaha? | Owabazaire bange………………..…...1  Owa ise zara nyowe…………………..2  Na I banyowe………………………..3  Na aboruganda bange………………..4  Nyenka………………………………..5 |  |

**Ekikcheka kya kabiri; Hati kankuhabuze ebitukwatana na omwana onu (baby’s name) (Taa emanyikirizo emu ha esura emu yonka ha ebicaguzo 9-16b. Guruka 16b kasangwa 16a egarukirwemu)**

| 10 | Baby ine obukuru ki?  **Handika obukuru mu myeezi** |  |  |
| --- | --- | --- | --- |
| 11 | Okamuzara di? |  |  |
| 12 | Ekikura kye | mwoojo……………………………1  mwisiki…………………………….2 |  |
| 13 | Baby okamuzara omu? | Nangwa……………………………1  Ego ……………………………....2 |  |
| 14 | Boona baroho/ bomeezi? | Nangwa……………………………1  Ego …………………………….....2 |  |
| 15 | Baby onu, okamuzara ha meezi aingaha? | Mwenda………………………..........1  Munana ……………………….........2  Musanju……………………………3 |  |
| 16a | Akazarwa ne’ratiri zingaha ? | ≥ 2500gms…………………………1  ≤ 2499gms…………………............2 | *Kebeera mu passipota/ ekipande* |
| *16b* | Mukurora kwawe, omana onu akaba no bulemezi ki? | Mukooto ……………………………...1  Hagati aho……………………………2  Katito ………...………………………3 | *Guruka mama bwaraba ina passipota/ ekipande* |

**Ekichweka kya kasatu; ebikwata na ha’buhereza nenkozesa ye’ ebyobomezi (Taa emanyikiro emu buli kikkaguzo)**

| 17 | Okakebezaho enda egyo? | Nangwa………………………….…..1  Ego ………………………….……...2 | *Kasangwa nangwa genda ha Q.21* |
| --- | --- | --- | --- |
| 18 | Bakagikebera nkaha? | Hairwaro lya gavamenti..……………..1  Mu puraiveti…………………….........2  Owa TBA………….…………….……3 |  |
| 19 | Okagyayo emirundi ingaha? | Guumu.…………………………….….1  Ebiri ………………………………….2  Esaatu ………………..…………….…3  Eena ………………………………….4  Etaanu +………………………………5 |  |
| 20 | Baby onu okamuzaalira nkaha? | Irwaro lya Govt ………………………1  Mu kilinic……………..………...……2  Mu puraiveti………………………….3  Nta’zaliire mu irwario………….…….4 |  |
| 21 | Kinu kya abazalire omuka boonka, okaba oina mama kit? | Nangwa …………………………….1  Ego ……………………….................2 |  |
| 22 | Okazara ota? | Kurungi………………………..…….1  Bakansemeza ………………………..2 |  |
| 23 | Okamarayo ebiiro bingaha mu kicweka eki wazaliremu? | Nkatahira ho………………………..1  Ekiiro kiimu………………………….2  Ebiiro 2 to 4 ……………….................3  Ebiiro 5 to 10 ……………….………..4 |  |
| 24 | Ekyaro kyawe kigira omujanjabi wa hakyaro rundi VHT? | Nangwa ………………………...……..1  Ego ……………………………..…....2 | *Kasangwa nangwa genda ha Q.30* |
| 25 | VHT akakubungiraho ozaire? | Nangwa ………………………………..0  Ego …………………………………....1 |  |
| 26 | Akakubungira emirundi ingaaha? | Gumu ……………………………….…1  Ebiiri ………………………………....2  Esaatu ………………………………….3  Kukira emirundi essatu …………….…4  Atambungire ………………………….5 |  |
| 27 | Harunungo rwa VHT akakusomesaho emiringo yo kulinda enkerembe? | Nangwa …………………………..1  Ego ……………………………....2 | *Kasangwa nangwa genda ha to Q.30* |
| 28 | Mukabaaza haali biiki na VHT?  **(teeka emanyikiro ha byoona ebigambirweho)** | Omulindo gwo okwonkya……………..1  Omulingo kwo okutagasa baby……….2  Okulinda omukundi mubuyonjo ………3  Ebyokugemesa enkerembe….…………4  Kurara hansi ya akatimba ka’miibu…………………….……….…5 |  |

**Ekichweka kya kaana: Endoleera ya enkeerembe eya’kazalibwa ( habwa ebikaguzo 30-33, ta emanyikirizo emu bulikikaguzo. Habwa ebikaguzo 29na 34, ta emanyikirizo buli ansa ehairwe)**

| 29 | Mu sabiti emu no’kyazaire halo omuntu weena ayakozere biinu?: | \| Kebera okwokya kwa baby \| 1 \| 2 \| \| --- \| --- \| --- \| \| Yakugambireho ha’enyonkya \| 1 \| 2 \| \| Yaku rozera no kuhabura no yoonkya? \| 1 \| 2 \| \| Kupiima ratiiri za baby? \| 1 \| 2 \| \| Ke’ebeera omukundi gwa baby? \| 1 \| 2 \| \| Ya’kusomeseza rundi kugambireho ha bubonero bya akabi mu’nkeremebe? \| 1 \| 2 \| |  |
| --- | --- | --- | --- | --- | --- | --- | --- | --- | --- | --- | --- | --- | --- | --- | --- | --- | --- | --- | --- | --- | --- |
| 30 | Oha yakujanjabire no’kyazaara? | Nyakaitu …………………………......1  Nyiina nyowe………….......................2  Nyiinezara nyowe……………………3  Muramukati wange……......................4  Iba nyowe……………….,…………..5  buusaho………………………………6 |  |
| 31 | Ayakujanjabiire aka kanga mu zinga ha? | Atasoome ………………………….1  Purimare…………………………….2  Secondare………………………..…..3  Ebyemirimo …………………….…..4 |  |
| 32 | **Kukwata ha’bakaazi boonka** omukaazi ayakujanjabire yaali azaireho rundiki ina abaana abe? | Nangwa………………………………1  Ego …………………………............2 | *Kasangwa nangwa genda ha Q.36* |
| 33 | **Abaana ina baingaha**? | 1  2  3  4  4+ |  |
| 34 | **Okahaburwaho o’mundolera yo mwana onu ebikwatanaho na binu?** | \| Okwonkya \| 0 \| 1 \| \| --- \| --- \| --- \| \| Okutagasa owana \| 0 \| 1 \| \| Endoleera y’oluleera \| 0 \| 1 \| \| Obunero obwakabi \| 0 \| 1 \| |  |

**Ekichweka kya kataanu; Endoleera yo mukundi ( habwa ebikaguzo 29-34, ta emanyikirizo emu bulikikaguzo)**

| **35** | Omukundi rundi orulera rwa enkerembe ruina okulindibwa ruta? | Otateire kintu kintu……………1  Ebindi…………………………2 |  |
| --- | --- | --- | --- |
| 36 | Oina ekintu kyona eki’wateire ha ruleera rwa baby onu rutakachwekere? | Nangwa……………………..0  Ego ………………………...1 |  |
| 37 | Okataho ki? | Spirit…………………………1  Obuusa ………..……………2  Itaaka ……………………..3  Omunyuuli………………….4  Ebyekisaa….………………..5 |  |
| 38 | Oha’yakuhaire ago amagezi? | omusehenyi………………......1  munyaruganda wange..............2  VHT…………….……………3  Buusaho………………………4  abandi……………………….. |  |

**Ekichweka kya mukaaga; Okutagasa enkerembe ( Habwa ebikaguzo 40-42 ta emanyikirizo emu bulikikaguzo. Habwa ebikaguzo 39, teeka emanyikirizo buli ansa ehairwe)**

| 39 | Enkerembe o ina kutagasibwa ataa? | Kumufumbata.....................................1  Kumusubika mu ebibande, engoye..................................................2  Ebindi....................................................3 |  |
| --- | --- | --- | --- |
| 40 | Okakoraki kutagasa baby onu nakyali enkerembe? | Nkakajweka kandi nyamusubika kuruga hamutwe kuhika ha bigere..................1  Nyakafumbataga …………….............2  Nkamwikaza haizi nanyowe nakyazarwa...................………............3 |  |
| 41 | Enkerembe eya kazalibwa eina kwoga hahoire akasumi ki | Hanyuma ya esaha 24...........................1  Nakyazalibwa .......................................2  Ebindi....................................................3 |  |
| 42 | Iwe onu okamwogya harabireho akasumii ki? | Na kyazarwa........................................1  Ekiro kimu kirabireho/ saaha 24.........................................................2 |  |

**Ekichweka kya musaanju; Okwonkya enkerembe ( habwa ebikaguzo 43-52, ta emanyikirizo emu bulikikaguzo.)**

| 43 | Enkerembe nekya zalibwa eina kutandika kwonka di? | Nakyazalibwa/ rundi musaha emu nakyazalibwa ........................................1  Ebindi ....................................................2 |  |
| --- | --- | --- | --- |
| 44 | Kiiki ekiwabandize kuuha baby onu? | Eibeere ………………………..............1  Amaata ga’ensana…………..........…....2  amaizi……………………….........……3  Gulucosi………………………..........…4  Ebindi.................................................. |  |
| 45 | Beebi onu okamwonkya hahaireho akasuumi ki? | Dakiika asaatu zitakarabireho…..............1  Hagati ye dakiika 30 ne saaha emu.......................……….........……….2  Saaha ibiiri……………….............…….3  Saaha 6 ………………………...........…4  Hanyuuma ya’ekiiro kiimu …...........…..5 |  |
| 46 | Baby onu okamuha amaata gaabandiza kimu agaba ga kyenju? | Nangwa ………………………………..0  Ego …………………………..………...1 | *Kasangwa ego genda ha Q 45* |
| 47 | Kasangwa nangwa, habwaki otamuhaire amaata ago agabandiza kimu. (Aga kyenju**)** | Gakaasa machafu………………………..1  Muziro/ titubaha………………………...2  Abandoleraga ba kampamagezi ngu ntagamuha…………………………….…3 |  |
| 48 | Habwaki wa mazire esaahu zihingwire ha 24 otakonkeze? | Nkaba ntaina maata……………........….1  Nkaaba ntamanyire engeri yo kumwonkya……………………………..2  Nkaba nimuha ebyokunya biindi…...…..3  Enjara ekaba etakumuruma, atakucuura.............................…….......…4  Timanyire.…………………...............….5 | *Otakihabuza kasangwa beebi bamwonkeeze mu saha 24* |
| 49 | Habwaki wa omwana wabandize kumuha ebyokunya bindi bitali amaata geibere? | Amaata gakaba gaha………….........….1  Nukwo bamba bwiire..........….....…….2 |  |
| 50 | Oha aya kuhabwire atyo? | Dakitali........................……...........……1  Ayandoleeraga......……………..........…2 |  |
| 51 | Wa yonkyaga emirundi nka eingaha? | 4 …………………….........................…1  8-10 ………………………....................2  Bulli yakacuuraga…............…................3 |  |
| 52 | Nahati okyayonkya byonkya? | Nangwa ………………….......................1  ego …………………………..................2  nkumuha ne’ebyokulya.............…........….3 |  |

**Ekichweka kya munana : obuboneero obwakabi**

| 53 | Onyina obubonero obwa’kaabi byomanyire mu nkerrebe? | Nangwa ………………………..0  Ego …. ………………………...1 | *Kasangwa nangwa genda ha 53* |
| --- | --- | --- | --- |

54. Ngambiraho obubonero bwomanyire (teeka emanyikirizoha obubonero bwona obugambirwe)

| **Okabonero ka kaabi** | Ego |
| --- | --- |
| Enkeerembe eli ntaito munu | 1 |
| Enkeeerembe ekuura muno | 2 |
| Enkeerembe etakyonka | 3 |
| Omubiru/ ebigeere bye’enkeerembe biiri erangi ye’kyenju | 4 |
| Enkerembe ekwokya muno/ oruswijaswija | 5 |
| Enkeerembe ekufuca muno | 6 |
| *Enkerembe okuura etaina mani* | 7 |
| Enkeerembe ebyamirire muno/ okumwimukya kuli kuzibu | 8 |
| Okwesiika | 9 |
| Omukundi/ oruleera gukununka/ gwengerire | 10 |
| Enooga/ oruhere rulimu amasiira | 11 |
| Amaiso gengere/ galimu otusiira | 12 |
| Enkeerembe etakuhikya kurungi | 13 |
|  |  |

| 55 | Oha akuyamba mukuroleera baby onu? | Ise ……………………….……………1  Abazaire bange………………………..2  Bagenzi bange……………………….3  Aborugandabe……………………….4  Nyenka..….……………………………5  Others specify…………………….6 |  |
| --- | --- | --- | --- |

Tumalirize, oina ekikaguzo kyona ?

Caali webaale muno
